# Parliament and the Professions

**Published:** 1919-04-12

**Authors:** 


### PARLIAMENT AND THE PROFESSIONS.

### Military Massage Service.

Capt. Guest informed Colonel Yate that a form of petition has been received from members of the Military Massage Service. Full consideration has not yet been given to all the matters raised, but it has been possible already to deal favourably with some of them.

### Dr. Freyberger.

Colonel Burn asked the Home Secretary what was the act of disloyalty that caused the naturalisation certificate of Dr. Freyberger to be rescinded; and why he was employed officially as a pathological expert to conduct post-mortem examinations when many experts of purely British blood could have filled that position. Mr. Shortt replied that the act of disloyalty was the aiding and abetting of Dr. Freyberger's son in an attempt to disclaim British in favour of another nationality and so avoid military service. He believed that many years ago Dr. Freyberger's name was included by the London County Council in a list of competent pathologists whom London coroners were authorised to consult, but so far as Mr. Shortt was aware, he was never employed by the Government.

### Tuberculosis Inter-departmental Committee.

Major Astor stated that the number of discharged tuberculous men awaiting residential treatment on February 28 last was approximately 470. The President of the Local Government Board and the Minister of Pensions have appointed an Inter-departmental Committee to report on this subject, and on the reintroduction of the men into employment, especially on the land.

### Government Control of Hospitals.

Major Astor said that there is no foundation for the suggestions contained in a question by Major Lane Fox asking whether there is any truth in the report that it is the intention of the Government by successive steps to take over all hospitals during the next forty years—beginning at once with St. George's Hospital.

